# Rethinking the withholding/withdrawing distinction: the cultural construction of “life-support” and the framing of end-of-life decisions

**DOI:** 10.1186/s40248-015-0004-5

**Published:** 2015-03-17

**Authors:** Yechiel M Barilan

**Affiliations:** Sackler School of Medicine, Tel Aviv University, Tel Aviv, Israel

**Keywords:** End-of-life, Life-support, Technology-philosophy of, Withdrawing, Withholding

## Abstract

This paper is a theoretical and empirically informed examination of the naturalist distinction between withholding and withdrawing life-support.

Drawing on the history of mechanical ventilation and on a recent Israeli law containing a novel approach to disconnecting life-support at the end of life, it is argued that the design of machines predicates the division line between “active” and “passive” interventions, and that the distinction itself might be morally self-defeating.

Informed by insights from moral psychology, behavioral economics and philosophies of technology, the paper warns against the placement of this old distinction at the heart of the moral and legal regulation of life-support at the end of life.

## Background

This paper relies on insights from social psychology, science and technology studies, and behavioral economics in order to shed light on the cultural construction of the withholding/withdrawing distinction and its moral implications. I will argue against a reductionist understanding of normativity in which norms are functions that assign normative values (permitted/prohibited; obligatory/supererogatory) to given situations. Drawing on a recent Israeli legislation on withdrawal of life-support, I will show how the construction of medical technology and its regulation loom large over the framing of end-of-life decision making and its actual regulation.

In this article I rely on a tentative and intuitive definition of technology according to which "technology" is an algorithmic process, developed and used by humans with the intention of handling efficiently a targeted problem [1]. An algorithm is an impersonal (hence objective) process which is reducible to a sequence of simple and clear units which consequently guarantees a relatively reproducible and predictable results [2]. The bylaws of a bureaucratic system, as well as a motor car are two examples of technology. Mechanical ventilators are a technology because they guarantee gas exchange in the lungs by simple and reproducible steps and controls. This paper is concerned with the medical service of mechanical ventilation in the contemporary affluent West. This service is a complex technology encompassing machines and bureaucracy (i.e. bio-law).

## Main text

### The problem reformulated

Since its very beginning, the debate on withholding and withdrawing life-support seems to be about action, inaction and intention during a narrow window-time. This window-time is best captured when a healthcare professional disconnects a life-support from a still living patient.

But no less important is the question of policy making, which is not about the probity of particular actions, but on the social construction of the rules on withholding and withdrawing life-support and the circumstances in which such actions are contemplated. A reductionist understanding of moral (and other) laws would behold rules as instruments of specification, abstract functions that connect a prototypical situation (e.g. “a terminal patient who wishes to die in order to escape suffering”) to a directive (“you may, must or must not do A”) [3]. But laws cast shadows on other laws, on implicit norms, and on social circumstances. This observation is not limited to “deontology” in the sense of ethical doctrine, such as “Kantian ethics”, but to any norm that is (1) canonized (has an established verbal formulation), (2) formal (carries a normative authority in society) and (3) formulated in terms of permission, prohibition or duty. In this paper I wish to examine these indirect aspects of deontological normativity.

Let’s think of Roger, a sixty year old man, living in a Western democracy and suffering from a degenerative disease. Roger knows that when his condition deteriorates further he would have the legal power to refuse life-support, even at the risk of death. Roger also knows that if he accepts mechanical ventilation and later changes his mind and choose not to be ventilated, it may not be legal to act according to this choice. He will certainly encounter practical hurdles, such as elaborate legal steps and psychological evaluations; he may not have the physical, mental and social resources to cope and express his wish convincingly and effectively. If he is aware of the unfriendliness of his clinical, social and legal environment to disconnecting life-support, he would think that his own worries are opposed to social norms and had better be suppressed. He might particularly suppress the complex desire to be connected to life-support now (and be "burden on others") with the easy option of disconnecting later on. He might think that "a good patient" does not think this way. Hence, it is quite likely that Roger’s decision whether to accept life-support in the first place might be influenced by the actual availability of disconnection later on.

In a similar vein, consider an eighty five year old patient with acute pulmonary edema secondary to chronic heart failure. This is the third such episode for the particular patient in a year. The last event was marked by a difficult and long weaning from the ventilator. Suppose that the patient has the moral and legal power to refuse mechanical ventilation. Her choice should be influenced by her own personal experience of illness and her personal and communal values. But in actuality, choice is likely to be influenced by the structuring of respiratory care. If active disconnection of life-support is prohibited, this patient has to factor in the risk of irreversible mechanical ventilation that would go days on end, regardless of the possibility that a temporary extreme measure (i.e. life on mechanical ventilation) has become a permanent one. On the other hand, if active disconnection of life-support is possible and easily obtainable, especially when its nature has changed (i.e. from a temporary to permanent), the risk involved in acceptance of intubation is much smaller. If the situation does not improve within a few days, comfortable disconnection and easy death would be allowed. It is not possible to tell what choice this hypothetical patients will make; but it is possible to infer that some such patients will accept mechanical ventilation only if later disconnection is available for them; and that in the absence of such availability, they would rather accept immediate death to the risk of weeks of agony on mechanical ventilation. Moreover, they might feel even more comfortable and truthful if such choices were not constructed as personal preferences (e.g. "want"/"does not want" to live) but framed by objective and relevant factors as well, such as whether there are or are not reasonable chances for weaning and rehabilitation. For such patients, a prohibition on disconnecting life-support and the hostile cultural construction of decisions to disconnect are actually a life-shortening measure. It is a legal instrument which self-defeats its life protecting goal. On the other hand, a permission to actively disconnect life-support may actually serve the value of human life, because the freedom entailed may psychologically empower patients and doctors to try a little more therapeutic and palliative measures.

If one wishes to argue that the laws constraining euthanasia are not meant to protect life, but the "integrity" of the medical profession, one will have to defend a situation in which an avowedly altruistic profession grants higher priority to its own "integrity" than to patients' lives, dignity and freedom from suffering.

Regulation of life-support has direct and indirect dimensions. The direct one is the prescriptive power of the law; the indirect one is the impact of the law on actions that are either peripheral or remote from its scope. The impact of the prohibition on disconnecting life-support on the choice whether to accept life-support care in the first place exemplifies an impact on a peripheral choice. The public image of mechanical ventilation towards the end of life as a one way street is even more peripheral to the deontological problem “may or may not disconnect”.

In addition to the indirect impacts of one set of laws (e.g. right to demand discontinuation of life-support) on practice in a different context of action (e.g. whether one acts on one’s power to refuse life-support), the law, especially its moralizing messages, may indirectly affect the actual availability of a certain choice. One may formally have the right to disconnect from life-support, but lack of information, bureaucratic barriers, such as a long chain of evaluative and deliberative processes and an atmosphere of disapproval may dissuade patients, especially the weak and hesitant from initiating a process that will allow them to consolidate an informed choice and benefit from it without excessive burden.

We have pointed out two phenomena. The first is the impact of laws on behaviour not purportedly regulated by those laws; and the impact of circumstances on the actual implementation of the law. Additionally, reflection on a recent Israeli law may open our eyes to another layer of indirect regulation. It is the influence of the development of a certain technology in society on the construction of its meaning and goals. Laws, sentiments and attitudes are modifiable; some structural factors, such as historical circumstances are not. Our analysis of the Israeli law will pave the way for an understanding of the roles of such structural factors in actual decision making in healthcare and how people and society cope with these factors.

### The Israeli 2005 Law on the patient nearing death

In 2005, The Israeli Parliament enacted the Law on the Patient Nearing Death. The law was drafted by a public committee chaired by a physician, rabbi and ethicist, and whose goal was to seek the broadest possible consensus on the regulation of end-of-life medical decisions [4,5]. In order to circumvent the action/inaction as well as the direct/indirect distinctions, the committee adopted the concepts of “discrete” and “continuous” modalities of life-support. Dialysis and blood transfusion are paradigmatic of discrete treatments, since they are comprised of distinct sessions with intervals in between. A decision to discontinue dialysis is a decision that entails inaction. Following the recommendations of the committee, in line with Jewish law and many other moral doctrines, the law empowers patients to discontinue “discrete” treatments. This means that the patient has the power to choose not to undergo dialysis anymore. The law also prohibits “direct, active and deliberate*”* disconnection of continuous life-support, such as mechanical ventilation. So far, there is nothing new about this act of legislation. But the committee and the law have gone two steps further. First, the law ordains that a continuous treatment that has become discrete may be discontinued. Second, by explicitly mentioning the possibility of a continuous action becoming a discrete one, the law creates a new psycho-legal entity and endows it with realism and probity. The law has given engineers, clinicians and administrators a new concept to think about, a goal to achieve. Additionally, the lawmakers had a specific idea in mind.

In 1978, the general manager of a religious Jewish hospital presented its rabbi with the idea of attaching timers to respirators. The timers would be reset regularly until it is decided that life-support be discontinued. In this case, caregivers would refrain from resetting the timers, thus allowing mechanical ventilation to cease without active intervention. Religious Jews use such timers in order to control electrical appliances, whose active operation is prohibited on the Shabbat. In line with Jewish law’s teaching that at least “in times of necessity”, indirect and passive action might be permissible as a means to bypass a prohibition, the rabbi endorsed the device. But nothing has been done about it. Almost thirty years after the event, the committee extracted this idea from the rabbinic literature and incorporated it in biomedical regulation. Following the enactment of the Patient Nearing Death Act in 2005, the Minister of Health nominated another committee to oversee the development and implementation of such a device. A few weeks ago, the IRB of Hadassah Hospital in Jerusalem approved a pilot study of a respirator connected to a timer.

The transformation of ordinary respirators to timers-dependent devices poses a formidable technological and regulatory challenge. Owing to their status as “life-support”, these machines are designed with the highest levels of safety possible, in ways that render automatic, accidental or passive stoppage quite impossible. Conversely, the concept of the timer-dependent respirators requires routine active human intervention in order to keep them going, while prudential risk management seeks to eliminate the dependence of safety on human intervention. Put in other words, the safety of most respirator-dependent patients is disposed against the instillation of timers; while a bioethics-friendly technology might prefer this option for passive discontinuation. The two conceptual approaches are doomed to collide: if we wish to render life-support treatment fully conscientious, we must allow people to choose disconnecting it; if we wish it to be safe, we must set technical and regulatory hurdles and limits on discontinuation. In fact, society shows very low level of tolerance to accidental loss of patients’ lives, especially in situations such as elective surgery, regulation inevitably creates difficulties for the few patients who wish to die. In a bioethical utopia, all those who wish to stay on life-support will not suffer accidental stoppage, and all those who wish to die, may have the power to autonomously choose so in the appropriate condition and situation. The bioethical, clinical and legal communities are entangled with the casuistic debate on withholding/withdrawing, not realizing that their two major strategic goals – to preserve life and to empower incurable patients who suffer terribly to make decisions regarding their life-support – are not fully compatible with each other.

We may observe a pattern. The pumping and gas-exchange related technology of the respirator is not an applied technology as such. But life-support had stabilized as a kind of an applied technology and reached a closure as a solution to the challenge of full anesthesia and polio. This closure is the construction of respiratory and life-support care as a distinct clinical service along with a set of habits, rules, values, and sets of experts that monopolize its use. But this closure was unravelling when life-support technologies were incorporated in the care of acute and deteriorating patients began to gain currency. This turn in the history of life-support technology called for an interpretive flexibility that will allow a redefinition of the problems this technology is set to answer [6].

Indeed, it is about time we ask ourselves what is the chief goal of mechanical ventilators; how we wish to conceptualize them as “clinical instruments”. One answer might be that mechanical ventilators are primarily life-saving devices and, consequently, they must be engineered with commitment to maximum safety. Within such a framework, clinical trials and implementation of timer-dependent machines would be ethically problematic. But a different answer might be that mechanical ventilators are transitional supportive devices, whose chief goal is the temporary stabilization of patients until definite decisions of care can be made. Within this conceptual framework, ventilators must not be designed in manners that render choices of discontinuation psychologically as well as practically difficult. Hence, the instillation of timers actually corrects an engineering bias towards maximum mechanical safety at the expense of effective autonomous choices about care. A third answer, yet, would wish to divide patients into two groups, those who fit in the first conceptualization of life-support (e.g. patient undergoing elective surgery); and those who fit the latter (e.g. the hypothetical patient, Roger, discussed in the beginning of this article). Such a division might allow a step-wise decision making on life-support: patients may be first placed on ordinary life-support; then, upon their informed request be transferred to a timer-dependent life-support; and only then, passive discontinuation may be chosen (or not). Patients might even wish to switch back from choice-oriented machines, the timer-dependent ones, to safety oriented machines (the ordinary ones). Such a choice might appear hypothetical, but it is also likely that at least for a certain period of time (e.g. when a child’s wedding is approaching) patients might wish to spare themselves the mental stress of choosing daily whether they wish the timers be reset. On the other hand, the division of patients on ventilators into distinct groups may raise a worry that “patients on times” might be discriminated against in terms of resource allocation (e.g. a bed in intensive care; queuing for an expensive procedure), even before they have expressed a wish to die.

This is not a mere exercise in casuistic thought-experiments. In the 1970s, Tversky, Kahneman and other scholars in psychology and economics have coined the term “framing” in relation to the verbal or situational presentation of a practical problem. They have showed that people’s choices are influenced by the framing of a problem regardless of its actual content. For example, many more people would consent to donate organs if you tell them that organs would be harvested unless they refuse in comparison to the numbers of people who would donate organs actively. The former is known as the “opting out” choice, whereas the latter is known as “opting in”. In both modes of framing, the content of the decision is identical (i.e. whether organs will be harvested or not) and the liberty of the agents equal (i.e. freedom from either coercion or sanction, and awareness of this freedom), but people’s actual choice seem to reflect the framing, as if a side issue (the choice of words, however synonymous semantically) bears on choice [7].

It follows, that if we design respirators and represent them as safety-oriented machines, choice of discontinuation will thus be framed as an exceptional deviation from default, "good" practice. A moderate attitude will accept such a choice pending elaborate procedures (e.g. psychological evaluation, juridical ruling); a stringent approach might regard such a choice as falling outside the scope of biomedicine and the integrity of the virtuous physician. But if we design respirators and represent them as a choice-oriented approach that prevents patients from dying before a fully informed and conscientious decision is made about their care, then, disconnecting certain patients from life-support would appear as natural as hooking them onto the machines in the first place.

An additional complication is the “identifiable victim effect” [8], according to which, people tend to exert greater efforts in the protection of the life of a known person (e.g. the patient Roger), than a generic person. More efforts will be directed to protect Roger, rather than to prevent one accidental death of a yet unknown patient on a mechanical ventilation. In my view, this effect, or a similar psychological process, has pushed the Israeli legislators to accommodate the few patients who wish to disconnect from life-support at the possible expense of risking compromised patient safety. Even though the patients who wish to disconnect are not “identifiable” as specific individuals, the ethical debate on disconnecting life-support and the motivation to solve the plight of these patients, frames the debate on a manner that renders them “identifiable” while inadvertently relegating all other patients to the periphery of the regulators’ attention.

The same law that sanctioned the timers-dependent ventilators also states that the physician has to comply with “good clinical standards of palliative care… according to the circumstances” (clause 23a). Owing to the opposition of the ministry of finance, the law does not contain an explicit right to hospice care^a^. Even though the allocation of health-resources and discontinuation of life-support are two distinct regulatory domains, one may worry about the possibility that some patients would not choose to disconnect if they were given hospice care. Not only is this one more example of the indirect impact of the law in a self-defeating manner (i.e. someone will choose to die and will die, even though it is possible to help his to go on living), but it also shows the power of the law to influence reality by its structure and level of specificity. Whereas, the Israeli lawmakers choose to define the right to palliative care in a general and conditional manner, the attention given to disconnection of life-support is specific and operational. Human values, the morality of discontinuation of life-support is delegate to the technology of timers [9]. Additionally, whereas the words of the law have been formulated so as to invite a specific technology, the term "hospice" is deliberately absent from the law's horizons. The R&D associated with timer-dependent respirators imply financial power; the refusal to grant an explicit right to hospice care entails the exclusion of the disempowered. The combined possible effect of the clause in the law on life-support technology with the silent clause on hospice care might be the development of an option for the poor to disconnect and die, but not to receive hospice care.

How can we know that the broader framing of a medical service is at the heart of the matter, rather than the naked distinction between “active” and “passive” action?

For many years already hospitals use what the Israeli legislator would later define as “discrete life-support”. When patients need transport (e.g. from the floor to the CT (Computed Tomography), or from one room to another), they are switched from electricity-powered ventilators to pressure powered ones (also known as POPV ventilation). The latter depend for energy on the high-pressure in the oxygen tank and not on AC electricity, thus allowing freedom of motion. Since such ventilators require change of oxygen-tanks, they are practically a sort of discrete life-support. Patients may choose to hook on pressure-dependent machines with the liberty to refuse change of balloon whenever they please. However, even though experienced clinicians participated in the committee drafting the law, the use of pressure-powered machines was not discussed at all. I conjecture that the reason for this omission and the consequent effort at developing a novel timer-dependent respirator is psychological. Because the pressure-dependent machines are already construed and received as belonging to the “life-saving” category, harnessing them in the service of discontinuing life-support might create a cognitive dissonance. Moreover, since the only modification needed is switching off safety features (e.g. the alarm), we are psychologically less inclined to think about such intervention. But the experimental new respirators are not construed on the downgrading of safety but on a design that adds something - a timer to the already safe machine. It seems that in response to a new problem people tend to design a new technology rather than redefine the uses of an existing technology that by sheer accident can solve the problem. The new Israeli law finds the answer to a clinical problem “in a specifically designed technology” rather than by having the problem solved or dissolved through “rethinking” existing objects and circumstances. It is easier to conceptualize questions at the concrete level (e.g. how to let life-support cease without direct action) than at the level of comprehensive meanings and goals (e.g. what is the role of life-support in the care of incurable patients? what is “good death”?).

We may further observe that even though the withdrawing/withholding distinction seems naturalist and straightforward, it is actually leaning on perceived realities rather than on solid evidence^b^ [10]. We have just seen that the related categories of “discrete” and “continuous” care suck their meaning from the framing of the context of care and its goal. We will now see that the taken-for-granted association of “active withdrawal” with causing death is unsubstantiated either.

Almost all patients on mechanical ventilation do have a residual capacity for independent breathing. Some are likely to die within minutes or hours of discontinuation; others might not. We had better remind ourselves of the famous case of Karen Ann Quinlan, whose doctors swore under oath that she would die if disconnected from life-support. After the court ruled in favour of active disconnecting, she survived nine more years. If we behold patients like Quinlan from the prism of tort and criminal law, her death might be construed as direct outcome of either negligent care or malice. But if we behold this very same choice and action from the perspective of clinical care, we understand that (with the exception of patients undergoing general anesthesia) patients do not die immediately after his or her respirator is disconnected; and that clinicians’ prognostication regarding post-disconnection survival is far from being dependable. It is not unlikely that a little clinical humility might extract many clinicians from the withdrawing/withholding dilemma and save many patients the agonies of unwanted suffering and loss of control over their care.

Historically, mechanical ventilation was introduced as a means to keep alive patients afflicted by polio and patients undergoing general anesthesia until their paralysis is resolved. In this context, safety is a crucial property of life-support, because mechanical failure might directly and quickly bring about the death of patients who are likely to enjoy many years of good life [11]. But had the history of life-support taken a different track, if mechanical ventilators had been first developed as instruments of palliation, for example, then, lack of an easy way out, would have been considered a major flaw. In the same vein, if it were possible to administer life-support by discrete doses, like dialysis sessions, the withholding/withdrawing distinction would be much less relevant. The emergence of life-support as an aid to healthy people with transient paralysis, the continuous nature of mechanical ventilation and the structure of tort and criminal law that set high standards of responsibility on its disconnection are examples of three different structural factors in the moral problem of discontinuing life-support from incurable and willing patients. A structural factor is a variant which bears on a practical domain of life, even though the relevant aspects of this variant have been determined by considerations and circumstances that are unrelated to the problem in hand. Structural factors frame moral (and other practical) problems in ways that may undermine the agents’ capacity to act on what the agents consider as relevant values. The “problem in hand” in the ordinary regulation of life-support is whether and how Roger’s physicians may disconnect him from life-support; the structural factor is the simple fact that in order to die, Roger needs active intervention, while another patient may be at the very same clinical situation, and, yet, may need only an act of omission in order to let go and die; the “externality”, the unaccounted for moral price, is the impact of both (the regulation of disconnecting life-support, and its structural factors) on Roger’s choice whether to accept mechanical ventilation in the first place. Ideally, only clinical considerations (e.g. prognosis in terms of survival, quality of life and burden of care) should be taken into account when Roger makes this decision. But in actuality, the ethical, legal and administrative constraints play crucial roles in Roger’s decision making process. We can discern this pattern in Roger’s choice against his own life and with compromised autonomy. It is a choice against life, because he will die despite his willingness to try and live longer; it is carried out of compromised autonomy because his freedom to choose now is cancelled out by his lack of freedom to choose in the future. The recent Israeli Law is an interesting exercise in the deliberate alteration of the structural factors that corner the medical community to grapple with the withdrawing/withholding dilemma.

Interestingly, some of the first systems of artificial respiration relied on human labour, on assistants who would pump paper bags filled with air into patients’ lungs [12]. In disaster medicine as well, owing to lack of either machines or electricity, many patients are manually ventilated (bag-valve-mask ventilation). This kind of life-support may be considered “discrete” because every act of pumping is, principally, the product of deliberate human will. Moreover, since human hands tire often, the change of hands is another discrete step in the process of what the patient experiences as continuous ventilation. It follows that the notion of “active disconnection of life-support” is a side effect, an artifact of automatization of transferring action from human agency to dumb machines. Even though this automatization is artificial, it is not necessarily "unnatural", because the natural act of breathing goes without conscious attention; and it is impossible for people to voluntarily terminate breathing and suffocate to death.

In the next and last section we will see how the automatization and mechanization of life-support has diverted our moral focus from patients’ agency to the doctor-machine interface (i.e. withdrawing vs withholding/discrete vs continuous).

### The silencing power of framing

Virtually all legal systems that allow potentially lethal choices of no-treatment posit a thorough process of patient evaluation. Before a patient is allowed to die, expert must sign that they have examined the case well and they confirm the medical diagnosis, prognosis, mental lucidity and lack of untried or promising measures of palliation. These measures have been instituted in respect of both life and autonomy, as regulators and care-givers wish to make sure that choice has been freely made in a state of adequate understanding and mental powers.

However, an odd feature of the Israeli law on the patient nearing death act is the complete absence of such evaluations. In addition to the deliberate silence on hospice care (previous section), one may regard this lacuna as a disturbing silence on the assessment of choices against life. The law empowers every terminal patient to refuse life-support and life saving measures without any formal process of patient evaluation. Indeed, good medical practice requires proper evaluation regardless whether any law asks for it explicitly and regardless whether life is at stake or not. But since the Israeli committee and legislators focused their deliberation on the resolution of the withdrawing/withholding quandary, they ended up neglecting patient evaluation altogether. In this framework, which focuses on the physician-machine interface rather than on values and personal choices, doctors may rest assured that their professional integrity and personal conscience remain untainted by someone’s death.

Paradoxically, the human tendency to behold technology as a means of empowerment, control and certainty has led to the institution of technological barriers to human manipulation of life-support machines, thus rendering a choice to disconnect a complex set of deliberate actions. However, end-of-life and palliative care is less about control and certainty and more about choice, acceptance and ambiguity. The life-saving context of respiratory machines and the extraordinary commitment to avoid fatal accidents in elective surgery has rendered the design of life-support much less friendly to terminal care. Perhaps life-support machinery should redefined and redesigned in the context of palliative and end-of-life care. The Israeli reconceptualization and reshaping of respirators help doctors keep their hands clean from active disconnection; but a deeper and more comprehensive rethinking of life-support in palliative care should accommodate social and personal values more comprehensively and authentically.

Nick Clayton observes: "the Victorian litmus test of new technology was: "Does it answer"? In our time the question is more likely to be phrased: "does it work?" [13].

The apparent tragedy of mechanical ventilation of incurable patients is the perceived moral hurdle on shifting from a contemporary to a Victorian approach. We tend to think that if respirators work at the physiological-mechanical level, then it “works” as a clinical instrument even when it answers no clinical or other human need. If we find invasive care not being an answer to a clinical need, then its removal should be deemed a moral duty rather than a transgression of a taboo. Altering the technology might lower the hurdle; but a critical moral approach would wish to have the perception of a hurdle removed altogether.

## Conclusions

In this paper I have explored two hypothetical case scenarios as well as a recent and novel Israeli law on the end of life in an attempt to behold the withdrawing/withholding distinction in its broader psychological and historical contexts. We have seen that the distinction loses much of its moral appeal upon the realization that the extensive role of accidental and unrelated factors in the framing of certain interventions as either “active” or passive”. Moreover, we have also seen how a life-protecting moral tool may indirectly bring about unnecessary and unwanted loss of life.

**Figure 1:**
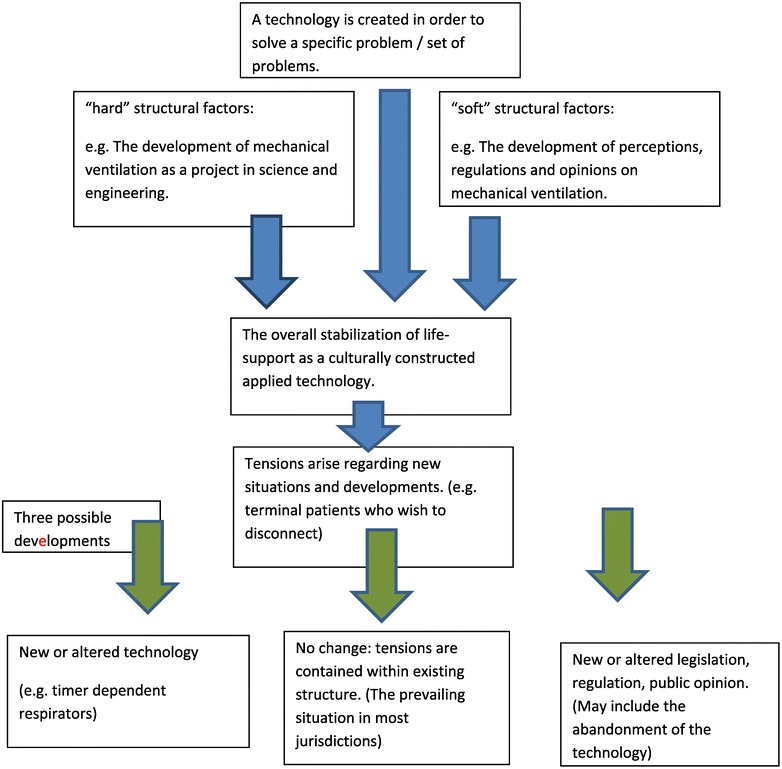
**Stabilization and revision of technology as a culturally constructed system.** (The case of mechanical ventilation).

A model for understanding the evolution of technologies as socially constructed systems is presented in Figure 1. Two kinds of structural factors influence (but not necessarily predicate) the consolidation of a technological service or system. The first is the “hard” ones, which are the given circumstances such as the consolidation of life-support as life-saving means with a predominant commitment to safety prior to the expansion of the use of life-support means towards intensive and palliative care. One manifestation of this structural factor is the built-in need for deliberate and elaborately active process of discontinuation of life-support. The second kind of structural factors, the “soft” ones, are the remediable formulations of the law, the structure of regulation and the public’s climate of opinion. The unintended impact of these hurdles on discontinuation of life-support on its acceptance in the first place is an example of these kinds of structural factors. Once a technology is socially constructed, and new problems emerge, such as the regulation of life-support of incurable and terminal patients, three pathways open-up. The first is the acceptance of the dictates of existing systems and regulation. This tracks offers these patients the rigid fail-proof service and ethos of “sanctity of life” that originated in the incorporation of mechanical ventilation in elective surgery and the support of polio patients. The second track is to come up with a novel technology so as not to collide with the legal and cultural constraints. This is the track taken by the Israeli law. The third, yet unchosen road, is to grapple with the clinical and personal needs of the new group of technology users (i.e. terminal and incurable patients) and use existing means as answers to “these” questions which have been deemed relevant, not others, which belong to different clinical settings and moral problems.

If we behold the problem of life-support at the end of life as determined by the interface between a human agent and a machine, the Israeli law has taught us that life-support, as well as other devices, may be redesigned in manners that reverse the meaning and implication of the withdrawing/withholding, active/passive as well as direct/indirect modes of action. But if we approach the regulation of life-support at the end of life from a humanistic perspective, we should grant less central roles to the given interaction between the human and the machine, and broaden the moral focus so as to encompass all relevant choices and actions, such as the acceptance of ventilatory care in the first place and the balance between security from mechanical failure and flexibility of choice.

## Endnotes

^a^A discussion in a parliamentary committee in which I myself participated as a representative of the palliative medicine chapter in the Israeli Medical Association.

^b^A “naturalist” distinction is a mode of reasoning common to universal human rationality, at least in the sense that it is found in most, if not all, normative systems and worldviews. Contemporary research has found that naturalist categories, such as the “action/omission” distinction is rooted in moral psychology and neurophysiology (e.g. [10]).
